# Knowledge, Attitudes, and Practices regarding Soil-Transmitted Helminthiasis among Village Health Volunteers in Nakhon Si Thammarat Province, Thailand: A Cross-Sectional Study

**DOI:** 10.3390/tropicalmed7020033

**Published:** 2022-02-19

**Authors:** Udomsak Narkkul, Prasit Na-ek, Jaranit Kaewkungwal, Chuchard Punsawad

**Affiliations:** 1 Department of Medical Science, School of Medicine, Walailak University, Nakhon Si Thammarat 80160, Thailand; udomsak.na@wu.ac.th (U.N.); prasit.na@wu.ac.th (P.N.-e.); 2 Research Center in Tropical Pathobiology, Walailak University, Nakhon Si Thammarat 80160, Thailand; 3 Department of Tropical Hygiene, Faculty of Tropical Medicine, Mahidol University, Bangkok 10400, Thailand; jaranit.kae@mahidol.ac.th

**Keywords:** soil-transmitted helminthiasis, village health volunteers, risk factors, Nakhon Si Thammarat province, Thailand

## Abstract

Soil-transmitted helminth infections are most prevalent in rural populations. Village health volunteers (VHVs) are the key individuals for Thai primary healthcare. Therefore, this study aimed to investigate the knowledge, attitudes, and practices regarding soil-transmitted helminthiasis in VHVs. A questionnaire survey was conducted among 552 VHVs randomly selected from four subdistricts in a southern province of Thailand. Sociodemographic variables and information regarding the knowledge, attitudes, and practices related to soil-transmitted helminthiasis were collected using a structured questionnaire. The results demonstrated that VHVs had poor knowledge (70.47%) and practices (66.49%); however, 69.57% had good attitudes. Most VHVs had inadequate knowledge and practices regarding soil-transmitted helminthiasis. VHVs who had been trained in parasitic infection control measures were 2.18 times more likely to have good knowledge. VHVs with a monthly family income of more than 307 USD were 1.58 times more likely to have a good attitude. VHVs with good knowledge were more likely to have good practices. In conclusion, the development of training programs and health promotion should be considered to enhance the knowledge, attitudes, and practices related to soil-transmitted helminthiasis in VHVs, who are the key individuals for providing health education to community members.

## 1. Introduction

Soil-transmitted helminths (STHs) are a group of parasitic intestinal worms that can infect humans through the ingestion of parasitic eggs or skin contact with motile larvae. Four STH species are of particular significance vis-à-vis public health: a roundworm (*Ascaris lumbricoides*), a whipworm (*Trichuris trichiura*), and two species of hookworm (*Necator americanus* and *Ancylostoma duodenale*) [1,2,3,4]. The World Health Organization (WHO) has estimated that approximately 1.5 billion people worldwide are infected with STH [5,6,7].

Helminth infections remain a significant health problem in rural communities in some regions of Thailand. A national survey conducted in 2009 reported that the prevalence of helminthiasis was 18.1% in the Thai population. Persistently high prevalences of opisthorchiasis and hookworm infections have been reported in the northeastern and southern regions of Thailand, respectively [8]. The high prevalence of intestinal parasitic infections is closely associated with poverty, climatic conditions, poor personal hygiene, poor sanitation, and unsafe drinking water [8,9,10,11]. Previous studies conducted in areas of southern Thailand have focused on the distribution of intestinal parasites in raw vegetables [12], adults [8], the elderly [13], school-age children [14,15,16], and village health volunteers (VHVs), who act as community health workers in the rural communities of Thailand [5].

VHVs are members of a Thai healthcare alliance that was established to promote healthcare service communication and collaboration at the primary level. In general, VHVs are local people who willingly volunteer to work for their communities on public health matters. Apart from their occupations, they generally act as mediators between local healthcare providers and the people. All VHVs receive information from public health personnel and function as key providers of information regarding health promotion and education, disease control, and the basic health services available to villagers [17]. Promoting an understanding of the knowledge, attitudes, and practices related to soil-transmitted helminthiasis among VHVs would be helpful in dissipating awareness about STH and the importance of personal hygiene, as VHVs aid the local healthcare personnel in providing health education to the community.

The availability of baseline data on knowledge, attitudes, and practices regarding soil-transmitted helminthiasis is necessary to plan appropriate control programs and strategies for VHVs. Therefore, this study aimed to investigate the knowledge, attitudes, and practices related to soil-transmitted helminthiasis in VHVs living in the Nopphitam District, Nakhon Si Thammarat Province, southern Thailand. The findings of this study will help strengthen the information currently available on knowledge, attitudes, and practices in relation to soil-transmitted helminthiasis, and can be used to encourage policymakers and public health officials to develop training programs for soil-transmitted helminthiasis and health promotion for community health workers.

## 2. Materials and Methods

### 2.1. Study Design and Setting

**Figure 1:**
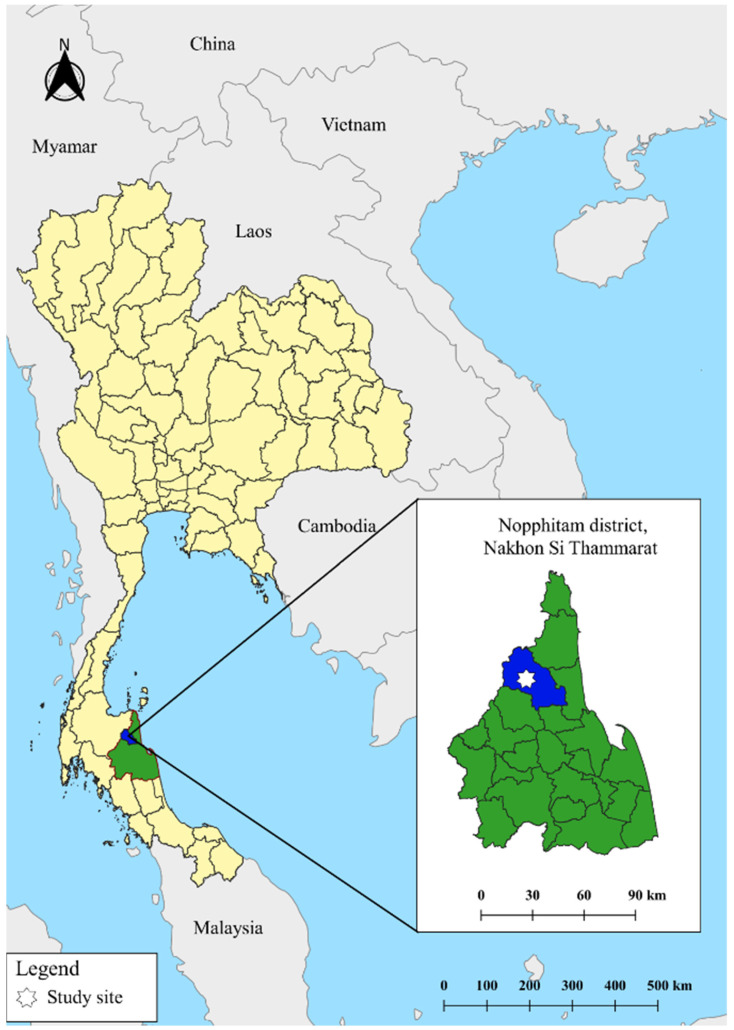
Location of the study site—Nopphitam District, Nakhon Si Thammarat Province, Thailand. Quantum GIS version 3.12 (ESRI base maps) was used to generate the map (https://qgis.org/en/site/ (accessed on 30 December 2021)).

This study utilized secondary data collected from a previous cross-sectional study on soil-transmitted helminthiasis among VHVs from October 2016 to September 2017 in the Nopphitam District, Nakhon Si Thammarat Province of southern Thailand. Nopphitam District is located at 8°43″ N latitude and 99°456″ E longitude, approximately 780 km south of Bangkok, Thailand’s capital (Figure 1). The prevalence of intestinal parasites in this area is 16% in children [16] and 9.3% in adults [5]. This district is divided into four sub-districts (tambon): Na Reng, Karo, Nopphitam, and Krungching. The geographical landscape of these four sub-districts is the same. There is only one community hospital in Nopphitam District, which is considered remote and rural. In this area, there are seven sub-district health promotion hospitals, including Ban Rong Lek, Ban Nob, Ban Lan Wua, Ban Tha Phut, Ban Huai Tong, Ban Pain, and Ban Hua Thung. The main access road is non-asphalt, whereas the remaining roads are asphalt. Most of the land is used as farmland and for rubber plantations. According to the Department of Provincial Administration, these districts had a total population of 33,561 in 2020. Agriculture is the main economic activity of the people in this area.

### 2.2. Study Population and Sample Size

The study was conducted among VHVs residing in four sub-districts of the Nopphitam District. This study aimed to investigate factors associated with a good knowledge, attitudes, and practices of STHs in VHVs. The sample size was determined using the single population proportion formula in the n4Studies application [18]. It was calculated by assuming the proportion of good knowledge, attitudes, and practices of STHs in VHVs to be 50% (*p* = 0.5), with a 95% confidence interval (CI; z = 1.96) and a 5% margin of error (d = 0.05). The sample size was calculated to be 385. We assumed that the final sample size would be reduced by 45% owing to incomplete data and non-response rate; therefore, we included all VHVs in this setting. The final sample size in this study was 558.

### 2.3. Data Collection and Questionnaire

A convenient sampling technique was used in the study. A structured questionnaire was developed and used to collect the data, which comprised two main sections (Supplementary Materials). The first section included the demographic and socioeconomic characteristics of the participants (i.e., age, gender, education level, and income). The second section included questions regarding the knowledge, attitudes, and practices related to soil-transmitted helminthiasis. All the questionnaires were checked for accuracy and completeness.

The knowledge level about soil-transmitted helminthiasis was assessed by requesting respondents to answer 20 questions about soil-transmitted helminthiasis (Supplementary Table S1) with response options comprising “Yes,” “No,” and “Not Sure.” The knowledge score was calculated as one for every “Yes” and zero for every “No” or “Not Sure.” The total score ranged from 0 to 20. Respondents with scores of more than 80 percent were considered to have good knowledge about soil-transmitted helminthiasis.

The attitude toward soil-transmitted helminthiasis was determined using a questionnaire containing 21 questions. A 3-point Likert scale was used in assessing the answers of this questionnaire, with three points awarded for an “Agree,” two points for ”Not Sure,” and one point if the answer was “Disagree”—for positive questions. A reversal in point rewarding was followed for the same answers with negative questions. The total scores ranged from 21 to 63. Respondents with scores greater than 80 percent were considered to have a good attitude.

The practice level related to soil-transmitted helminthiasis consisted of a 3-point Likert scale, which was determined using a questionnaire containing 20 questions. In the positive questions, three points were awarded if the participant answered “Usually,” two for “Sometimes,” and one if the answer was “Never.” In the case of negative questions, a reversal in point rewarding was followed for the same answers. The total score ranged from 20 to 60. Respondents with scores greater than 80 percent were considered to have good practices.

### 2.4. Statistical Analysis

Data were entered into an Excel database and subsequently double-checked for validation before analytical processing. All data analyses were performed using Stata/SE 14.0 for Mac. The survey data were analyzed for both descriptive and inferential statistics. Continuous variables, including knowledge, attitude, and practice scores, are described using mean and standard deviation (SD). Independent categorical variables, including demographic and socioeconomic data (i.e., age, education level, and income) and information on knowledge, attitudes, and practices of soil-transmitted helminthiasis are described using frequency and expressed as percentages. A mixed-effects logistic regression model with random effects at the sub-district health promotion hospital level was used to investigate the associations between outcomes and potential risk factors. Univariate analysis was used to examine the crude odds ratio (COR) of the binary outcome variable for each independent variable. All variables in the univariate analysis were subjected to multivariable analysis to adjust for possible confounders, by calculating adjusted odds ratios (AORs) with 95% CIs. Statistical significance was set at *p* < 0.05.

## 3. Results

### 3.1. Sociodemographic Characteristics

A total of 552 participants (68 men and 484 women) were enrolled in the study. The age group distribution showed that the majority (87.9%) were in the 31–60-years age group. Most of the participants (99.8%) were Buddhists. Most participants (85.3%) were married, and the majority (79.7%) were farmers. About half of the participants (55.4%) indicated that secondary education (high school) was the highest level of education they had received, and approximately one-third (33.3%) indicated that primary education was the highest level they had received. The average monthly household income of most participants (68.1%) was more than 307 USD. Having more than two family members was reported by 87.9% of the respondents. The majority of participants (46.2%) had 1–2 domestic dogs in the household, and most of them (53.5%) had no domestic cat at home (Table 1). Regarding VHVs’ experiences in parasitic control measures, most participants (57.1%) responded that no campaign for the prevention and control of parasitic infections existed in the community. Most participants (76.1%) had been trained in parasitic infection control measures. More than half of the participants (64.1%) had been advised to check stool samples for the diagnosis of parasitic infection, whereas half of the participants (52.7%) had no history of stool examination for parasitic infection in the past year. Further, most participants (85.9%) had not used any deworming medication in the past six months (Table 2).

### 3.2. Knowledge, Attitudes, and Practices Regarding Soil-Transmitted Helminthiasis

The average scores for knowledge, attitudes, and practices regarding soil-transmitted helminthiasis among VHVs are shown in Table 3. The average scores for knowledge, attitudes, and practices regarding soil-transmitted helminthiasis were 12.33, 52.29, and 46.28, respectively. The results revealed that 163 respondents (29.53%) had good knowledge of soil-transmitted helminthiasis, whereas 389 (70.47%) had poor knowledge. A total of 384 respondents (69.57%) had good attitudes, whereas 168 (30.43%) had poor attitudes toward soil-transmitted helminthiasis. Finally, 185 respondents (33.51%) had good practices, whereas 367 (66.49%) had poor practices related to the diagnosis and treatment of soil-transmitted helminthiasis.

### 3.3. Factors Associated with Knowledge about Soil-Transmitted Helminthiasis

The findings from the regression analysis performed to investigate the associations between knowledge about soil-transmitted helminthiasis and potential factors are summarized in Table 4. Univariate mixed-effect analysis of factors potentially associated with knowledge of soil-transmitted helminthiasis revealed significant associations between good knowledge and being trained in parasitic infection control measures. VHVs who had been trained in parasitic infection control measures were 1.99 times more likely to have good knowledge than those who had not been trained (odds ratio (OR) 1.99; 95% CI: 1.21–3.27). Good knowledge was not significantly associated with gender, age group, marital status, education level, occupation, monthly family income, family member, or ever having been advised to check a stool sample for diagnosis of a parasitic infection (95% CI includes 1).

Multivariate mixed-effect analysis of factors potentially associated with knowledge about soil-transmitted helminthiasis showed significant associations between good knowledge and being trained in parasitic infection control measures. VHVs who had been trained in parasitic infection control measures were 2.18 times more likely to have good knowledge than those who had not been trained (AOR 2.18; 95% CI: 1.26–3.79 after adjusting for other variables).

### 3.4. Factors Associated with Attitude toward Soil-Transmitted Helminthiasis

Findings from the regression analysis investigating the associations between the attitudes toward soil-transmitted helminthiasis and potential factors are summarized in Table 5. Univariate mixed-effect analysis of factors potentially associated with attitudes toward soil-transmitted helminthiasis revealed significant associations between good attitude and monthly family income, number of family members, and training in parasitic infection control measures. VHVs who had a monthly family income of more than 307 USD were 1.47 times more likely to have a good attitude than those who had a monthly family income less than or equal to 307 USD (OR 1.47; 95% CI: 1.01–2.17). VHVs who had more than two family members were 0.46 times less likely to possess a good attitude toward soil-transmitted helminthiasis than those who had less than or equal to two family members (OR 0.49; 95% CI: 0.26–0.94). VHVs who had been trained in parasitic infection control measures were 1.55 times more likely to have a good attitude than those who had not been trained (OR 1.55; 95% CI: 1.02–2.35). Good attitude was not significantly associated with gender, age group, marital status, education level, occupation, ever having been advised to check a stool sample for diagnosis of a parasitic infection, and knowledge level (95% CI includes 1). 

Multivariate mixed-effect analysis of factors potentially associated with attitudes of soil-transmitted helminthiasis revealed significant associations between a good attitude and monthly family income, number of family members, and being trained in parasitic infection control measures. VHVs who had a monthly family income of more than 307 USD were 1.58 times more likely to have a good attitude than those who had a monthly family income less than or equal to 307 USD (AOR 1.58; 95% CI: 1.05–2.39 after adjusting for other variables). VHVs who had more than two family members were 0.46 times less likely to have a good attitude than those who had less than or equal to two family members (AOR 0.46; 95% CI: 0.23–0.89 after adjusting for other variables). VHVs who had been trained in parasitic infection control measures were 1.68 times more likely to possess a good attitude than those who had not been trained (AOR 1.83; 95% CI: 1.03–2.76 after adjusting for other variables).

### 3.5. Factors Associated with Practices Related to Soil-Transmitted Helminthiasis

Findings from the regression analysis investigating the associations between practices of soil-transmitted helminthiasis and potential factors are summarized in Table 6. Univariate mixed-effect analysis of factors potentially associated with practices related to soil-transmitted helminthiasis revealed significant associations between good practices and ever having been advised to check a stool sample for diagnosis of a parasitic infection and knowledge level. VHVs who had been advised to check a stool sample for diagnosis of a parasitic infection were 1.62 times more likely to have good practices than those who had not been advised regarding diagnosis (OR 1.62; 95% CI: 1.11–2.38). VHVs who had good knowledge were 1.72 times more likely to have good practices than those who had poor knowledge (OR 1.72; 95% CI: 1.18–2.52). Good practices were not significantly associated with gender, age group, marital status, education level, occupation, monthly family income, number of family members, being trained in parasitic infection control measures, or attitude level (95% CI includes 1).

Multivariate mixed-effect analysis of factors potentially associated with practices of soil-transmitted helminthiasis revealed significant associations between good practices and ever having been advised to check a stool sample for diagnosis of a parasitic infection and knowledge level. VHVs who had been advised to check a stool sample for the diagnosis of parasitic infection were 1.57 times more likely to have good practices than those who had not been advised regarding diagnosis (AOR 1.57; 95% CI: 1.01–2.45 after adjusting for other variables). VHVs who had good knowledge were 1.75 times more likely to have good practices than those who had poor knowledge (AOR 1.75; 95% CI: 1.18–2.59 after adjusting for other variables).

### 3.6. Source Information on Soil-Transmitted Helminthiasis and Being Advised to Check for Parasitic Infections

**Figure 2:**
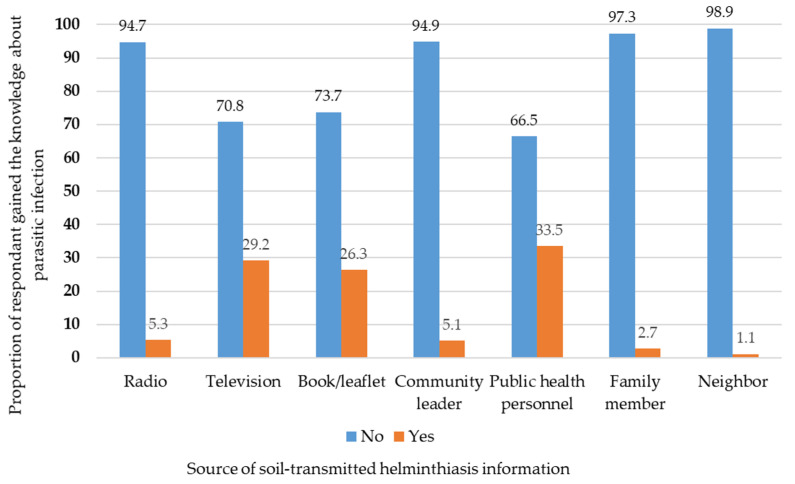
Proportion of respondents who gained knowledge on soil-transmitted helminthiasis by source.

**Figure 3:**
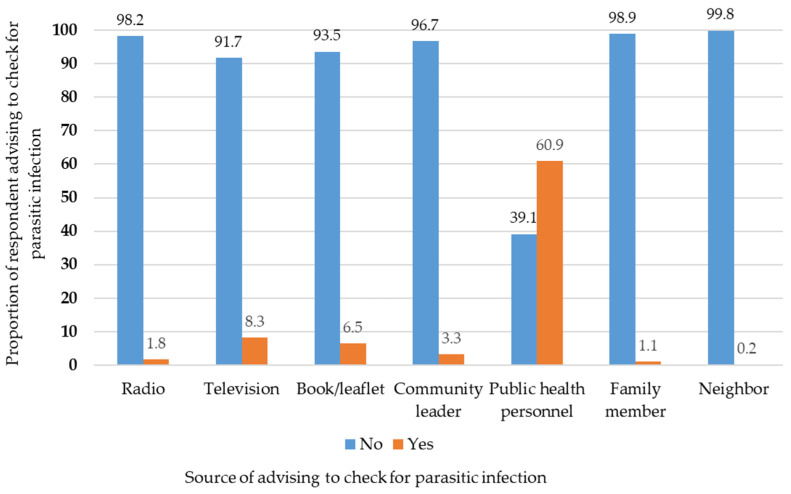
Proportion of respondents advised to check for parasitic diseases by source of advice.

Regarding the source of information on soil-transmitted helminthiasis, 33.5% of the participants had received information from public health personnel, followed by television (29.2%), books/leaflets (26.3%), radio (5.3%), community leaders (5.1%), family members (2.7%), and neighbors (1.1%) (Figure 2). Regarding the source of the advice to check for parasitic infections, most participants (60.9%) had gained the information from public health personnel, followed by television (8.3%), books/leaflets (6.5%), community leaders (3.3%), radio (1.8%), family members (1.1%), and neighbors (0.2%) (Figure 3).

### 3.7. Factors Associated with Source of Soil-Transmitted Helminthiasis Information

In terms of source of information regarding soil-transmitted helminthiasis, the results from univariate and multivariate mixed-effect analysis of factors potentially associated with knowledge regarding soil-transmitted helminthiasis revealed significant associations between good knowledge and public health personnel. VHVs who had gained knowledge about parasitic infection from public health personnel were 2.22 and 2.18 times more likely to have good knowledge than those who did not, respectively (OR 2.22; 95% CI: 1.43–3.43, AOR 2.18; 95% CI: 1.39–3.43; Table 7).

## 4. Discussion

This study was conducted in Nakhon Si Thammarat Province. STH infection is endemic to this area, according to old and recent studies and reports [5,8,13,16]. This study aimed to understand the knowledge, attitudes, and practices regarding soil-transmitted helminthiasis among VHVs. Our study demonstrated the importance of human demographics; information source for soil-transmitted helminthiasis knowledge; being advised to check for parasitic infections; and knowledge, attitudes, and practices related to soil-transmitted helminthiasis among VHVs.

The results of the knowledge, attitudes, and practices assessments related to soil-transmitted helminthiasis among VHVs showed that the average scores for soil-transmitted helminthiasis were low in knowledge and practices, whereas that of attitude was high. For the knowledge level, the results revealed that most respondents (70.47%) had poor knowledge related to soil-transmitted helminthiasis, which is consistent with a previous study conducted on pregnant women in Indonesia regarding knowledge about infection by an intestinal worm (43.9%) [19]. Similar findings have been reported in previous studies, showing that 4.1% of the respondents had accurate knowledge of the causes of STHs [20]. A low level of knowledge despite high awareness levels has been reported widely by knowledge, attitudes, and practices studies conducted in Bangladesh [21], Kenya [22], South Africa [23,24], and Ethiopia [25]. This could be due to a lack of knowledge regarding the causes, symptoms, transmission route, diagnosis, and prevention of soil-transmitted helminthiasis (Supplementary Table S1). Regarding the attitude level, most participants (69.57%) had a good attitude toward soil-transmitted helminthiasis. This finding could be explained by their knowledge on how to prevent soil-transmitted helminthiasis (Supplementary Table S2). In addition, the results showed that most participants (66.49%) had poor practices related to soil-transmitted helminthiasis. This result may be explained by bad habits, such as biting their nails, sucking their fingers, and defecating openly when they went out to work in the garden (Supplementary Table S3).

VHVs who had been trained in parasitic infection control measures were more likely to have better knowledge than those who had not been trained, after adjusting for other variables. This finding was similar to that of a previous study, which showed that people who gained knowledge, such as health education, increased their knowledge, attitudes, and practices [26].

VHVs with a monthly family income of more than 307 USD were more likely to have a good attitude than those with a monthly family income of less than or equal to 307 USD, after adjusting for other variables. This result was similar that of a previous study that showed that people in the income groups of 2000–4999 and 5000–9999 CNY held a more positive attitude than those who earned less than 1999 CNY every month [27]. This may be because a person’s financial status is linked to their education level. Other studies have found that people with higher education were more likely to gain better employment and consequently have higher monthly incomes [28]. In addition, people with higher incomes can afford to spend more money on personal protective equipment, such as masks [29], which indirectly reflects their more positive attitude. These two factors may explain the above results. VHVs who had more than two family members were less likely to have a good attitude than those who had less than or equal to two family members, after adjusting for other variables. Having a large number of family members results in many different effects, including the possible inculcation of a bad attitude among family members. VHVs who had been trained in parasitic infection control measures were 1.68 times more likely to have a good attitude. Our findings are in line with previous studies showing that those who had previously obtained knowledge, such as health education, might improve their knowledge, attitude, and practice scores [26].

VHVs who had been advised to check stool samples to diagnose parasitic infections were more likely to have good practices. Being advised to check stool samples to diagnose parasitic infections may be related to accurate knowledge overall, which should result in further improvements in practice. In line with the findings presented here, a survey in Sri Lanka found that individuals who had been advised on how to prevent STH infections demonstrated improved health practices [30]. VHVs with good knowledge were more likely to have good practices. Our findings are in line with those of a previous study that documented that knowledge is positively and significantly related to behavior [19]. Similar findings have been reported in many previous studies, showing that knowledge mediates the relationship between information and preventive behaviors [31,32,33].

The majority of VHVs had received their information regarding soil-transmitted helminthiasis from public health personnel, followed by television, books/leaflets, radio, community leaders, family members, and neighbors. This study shows a significant relationship between the major sources of information and knowledge. Our study revealed that VHVs who had gained knowledge about parasitic infections from public health personnel were more likely to be more knowledgeable than those who did not. Public health personnel are the most trusted sources of knowledge when compared with other sources. Our finding is consistent with prior research, which revealed that the most used and trusted information sources were the workplace, colleagues, television, and health workers [34].

This study has certain limitations. Regarding the study findings, it is important to consider potential methodological limitations, such as those normally observed in similar survey-based studies. First, the cross-sectional design of this study could only demonstrate associations between the different outcomes of interest and the observed determinants, and it is impractical to say anything about causality. Further, this study enrolled only VHVs in a particular region of southern Thailand. Additional studies should be conducted in urban or other rural areas and in different parts of the country.

## 5. Conclusions

The low levels of knowledge and practices observed in this study highlight the major deficiencies in current strategies. This study demonstrated critical needs: targeting health messages through public health officials to reach the most susceptible populations, developing training programs for soil-transmitted helminthiasis, and health promotion for VHVs in rural areas of southern Thailand. These findings could be useful in advancing awareness STH and the importance of personal hygiene, as they are commonly associated with groups of people who aid local healthcare personnel in providing health education to the community.

## Figures and Tables

**Table 1:** Sociodemographic characteristics of village health volunteers enrolled in the study.

Characteristics		Total (*n* = 552)	
		Number	Percentage
Sub-district health promotion hospital and community hospital			
	Ban Rong Lek	67	12.1
	Ban Nob	92	16.7
	Ban Lan Wua	73	13.2
	Ban Tha Phut	127	23.0
	Ban Huai Tong	32	5.8
	Ban Pain	84	15.2
	Ban Hua Thung	42	7.6
	Nopphitam hospital	35	6.3
Gender			
	Male	68	12.3
	Female	484	87.7
Age group			
	<30	40	7.2
	31–60	485	87.9
	>60	27	4.9
Religion			
	Buddhism	551	99.8
	Islam	1	0.2
Marital status			
	Unmarried	81	14.7
	Married	471	85.3
Education level			
	Primary school	173	31.3
	High school	306	55.4
	More than high school	73	13.2
Occupation			
	Not an agricultural worker	112	20.3
	Agricultural Worker	440	79.7
Income (USD)			
	≤307	176	31.9
	>307	376	68.1
Number of family members			
	1–2	67	12.1
	>2	485	87.9
Number of domestic dogs			
	No	237	42.9
	1–2	255	46.2
	3–4	51	9.2
	>4	9	1.7
Number of domestic cats			
	No	295	53.5
	1–2	192	34.8
	3–4	51	9.2
	>4	14	2.5

**Table 2:** VHVs’ experience with parasitic control measures.

Characteristics		Total (*n* = 552)	
		Number	Percentage
Had a parasitic infection campaign in the community in the last six months			
	No	315	57.1
	Yes	237	42.9
Trained in parasitic infections control measures			
	No	132	23.9
	Yes	420	76.1
Advised to check a stool sample for diagnosis of parasitic infection			
	No	198	35.9
	Yes	354	64.1
Had a stool examination for parasitic infection in the past one year			
	No	291	52.7
	Yes	261	47.3
Used any deworming medication in the past six months			
	No	474	85.9
	Yes	78	14.1

**Table 3:** Average scores, numbers, and percentages for knowledge, attitudes, and practices regarding soil-transmitted helminthiasis among VHVs.

Items		Total (*n* = 552)	
		Number	Percentage
Knowledge score (0–20) (Mean ± SD)		12.33 ± 4.89	
Attitude score (21–63) (Mean ± SD)		52.29 ± 4.76	
Practice score (20–60) (Mean ± SD)		46.28 ± 3.57	
	Poor	389	70.47
	Good	163	29.53
Attitude level			
	Poor	168	30.43
	Good	384	69.57
Practice level			
	Poor	367	66.49
	Good	185	33.51

**Table 4:** Univariate and multivariate mixed-effect logistic regression analysis of factors influencing knowledge.

Characteristics		N ^1^	Good Knowledge ^2^	OR ^3^ (95% CI)	AOR ^4^ (95% CI)
Gender					
	Male	68 (12.32)	26 (38.24)	Ref.	Ref.
	Female	484 (87.68)	137 (28.31)	0.68 (0.39–1.18)	0.75 (0.41–1.36)
Age group					
	≤30	40 (7.20)	13 (32.5)	Ref.	Ref.
	31–60	485 (87.90)	137 (28.25)	0.85 (0.42–1.73)	0.91 (0.44–1.86)
	>60	27 (4.90)	13 (48.14)	1.91 (0.68–5.40)	1.94 (0.63–5.97)
Married					
	No	81 (14.67)	23 (28.40)	Ref.	Ref.
	Yes	471 (85.33)	140 (29.72)	1.06 (0.62–1.82)	1.19 (0.68–2.07)
Education level					
	Primary school	173 (31.30)	48 (27.74)	Ref.	Ref.
	High school	306 (55.40)	92 (30.06)	1.08 (0.71–1.66)	1.25 (0.79–1.95)
	More than high school	73 (13.20)	23 (31.51)	1.07 (0.57–2.00)	1.28 (0.67–2.44)
Occupation					
	Not an agricultural worker	112 (20.29)	34 (30.36)	Ref.	Ref.
	Agricultural worker	440 (79.71)	129(29.32)	1.05(0.65–1.69)	0.96(0.59–1.57)
Income (USD)					
	≤307	176 (31.88)	53 (30.11)	Ref.	Ref.
	>307	376 (68.12)	110 (29.26)	1.01 (0.67–1.51)	1.05 (0.69–1.59)
Number of family members					
	≤2	67 (12.14)	20 (29.85)	Ref.	Ref.
	>2	485 (87.86)	143 (29.48)	0.92 (0.51–1.63)	0.94 (0.51–1.72)
Trained in parasitic infections control measures					
	No	132 (23.91)	25 (18.94)	Ref.	Ref.
	Yes	420 (76.09)	138 (32.86)	1.99 (1.21–3.27)	2.18 (1.26–3.79)
Advised to check a stool sample for diagnosis of parasitic infection					
	No	198 (35.90)	55 (27.78)	Ref.	Ref.
	Yes	354 (64.10)	108 (30.51)	1.09 (0.72–1.65)	0.83 (0.53–1.34)

^1^ Total sample of 552; ^2^ total good knowledge, 163; ^3^ odds ratio (OR); ^4^ adjusted odds ratio (AOR).

**Table 5:** Univariate and multivariate mixed-effect logistic regression analysis of factors influencing attitude.

Characteristics		N ^1^	Good Attitude ^2^	OR ^3^ (95% CI)	AOR ^4^ (95% CI)
Gender					
	Male	68 (12.32)	42 (61.76)	Ref.	Ref.
	Female	484 (87.68)	342 (70.66)	1.47 (0.85–2.52)	1.59 (0.88–2.85)
Age group					
	≤30	40 (7.20)	27 (67.50)	Ref.	Ref.
	31–60	485 (87.90)	339 (69.89)	1.14 (0.57–2.30)	1.11 (0.54–2.28)
	>60	27 (4.90)	18 (66.67)	1.01 (0.35–2.90)	1.01 (0.32–3.21)
Married					
	No	81 (14.67)	58 (71.60)	Ref.	Ref.
	Yes	471 (85.33)	326 (69.21)	0.86 (0.51–1.46)	0.97 (0.55–1.69)
Education level					
	Primary school	173 (31.30)	124 (71.67)	Ref.	Ref.
	High school	306 (55.40)	205 (66.99)	0.78 (0.52–1.18)	0.77 (0.49–1.19)
	More than high school	73 (13.20)	55 (75.34)	1.21 (0.64–2.30)	1.08 (0.55–2.11)
Occupation					
	Not an agricultural worker	112 (20.29)	79 (70.54)	Ref.	Ref.
	Agricultural worker	440 (79.71)	305 (69.32)	0.97 (0.61–1.56)	0.99 (0.61–1.62)
Income (USD)					
	≤307	176 (31.88)	113 (64.20)	Ref.	Ref.
	>307	376 (68.12)	271 (72.07)	1.47 (1.01–2.17)	1.58 (1.05–2.39)
Number of family members					
	≤2	67 (12.14)	54 (80.6)	Ref.	Ref.
	>2	485 (87.86)	330 (68.04)	0.49 (0.26–0.94)	0.46 (0.23–0.89)
Trained in parasitic infection control measures					
	No	132 (23.91)	82 (62.12)	Ref.	Ref.
	Yes	420 (76.09)	302 (71.90)	1.55 (1.02–2.35)	1.68 (1.03–2.76)
Advised to check a stool sample for diagnosis of parasitic infection					
	No	198 (35.90)	138 (69.69)	Ref.	Ref.
	Yes	354 (64.10)	246 (69.49)	1.01 (0.67–1.48)	0.74 (0.46–1.18)
Knowledge level					
	Poor	389 (70.47)	264 (67.86)	Ref.	Ref.
	Good	163 (29.53)	120 (73.61)	1.35 (0.89–2.05)	1.32 (0.86–2.03)

^1^ Total sample of 552; ^2^ total good attitudes, 384; ^3^ odds ratio (OR); ^4^ adjusted odds ratio (AOR).

**Table 6:** Univariate and multivariate mixed-effect logistic regression analysis of factors influencing practices.

Characteristics		N ^1^	Good Practice ^2^	OR ^3^ (95% CI)	AOR ^4^ (95% CI)
Gender					
	Male	68 (12.32)	25 (36.76)	Ref.	Ref.
	Female	484 (87.68)	160 (33.06)	0.83 (0.48–1.42)	0.82 (0.46–1.47)
Age group					
	≤30	40 (7.20)	10 (25.00)	Ref.	Ref.
	31–60	485 (87.90)	163 (33.61)	1.52 (0.72–3.19)	1.61 (0.75–3.46)
	>60	27 (4.90)	12 (44.44)	2.45 (0.86–7.03)	1.65 (0.53–5.09)
Married					
	No	81 (14.67)	27 (33.33)	Ref.	Ref.
	Yes	471 (85.33)	158 (33.55)	1.01 (0.61–1.68)	1.11 (0.65–1.90)
Education level					
	Primary school	173 (31.30)	6 7(38.73)	Ref.	Ref.
	High school	306 (55.40)	94 (30.72)	0.70 (0.47–1.03)	0.70 (0.46–1.05)
	More than high school	73 (13.20)	24 (32.88)	0.78 (0.43–1.40)	0.81 (0.45–1.48)
Occupation					
	Not an agricultural worker	112 (20.29)	37 (33.04)	Ref.	Ref.
	Agricultural worker	440 (79.71)	148 (33.64)	1.02 (0.65–1.59)	0.98 (0.62–1.54)
Income (USD)					
	≤307	176 (31.88)	66 (37.50)	Ref.	Ref.
	>307	376 (68.12)	119 (31.65)	0.77 (0.53–1.12)	0.81 (0.55–1.20)
Number of family members					
	≤2	67 (12.14)	28 (41.79)	Ref.	Ref.
	>2	485 (87.86)	157 (32.37)	0.66 (0.39–1.12)	0.66 (0.38–1.16)
Trained in parasitic infection control measures					
	No	132 (23.91)	36 (27.27)	Ref.	Ref.
	Yes	420 (76.09)	149 (35.48)	1.46 (0.94–2.25)	1.12 (0.67–1.86)
Advised to check a stool sample for diagnosis of parasitic infection					
	No	198 (35.90)	53 (26.77)	Ref.	Ref.
	Yes	354 (64.10)	132 (37.29)	1.62 (1.11–2.38)	1.57 (1.01–2.45)
Knowledge level					
	Poor	389 (70.47)	116 (29.82)	Ref.	Ref.
	Good	163 (29.53)	69 (42.33)	1.72 (1.18–2.52)	1.75 (1.18–2.59)
Attitude level					
	Poor	168 (30.43)	64 (38.10)	Ref.	Ref.
	Good	384 (69.57)	121 (31.51)	0.74 (0.51–1.09)	0.69 (0.47–1.03)

^1^ Total sample of 552; ^2^ total with good practices, 185; ^3^ odds ratio (OR); ^4^ adjusted odds ratio (AOR).

**Table 7:** Univariate and multivariate mixed-effect logistic regression analysis of factors associated with source of gaining knowledge about soil-transmitted helminthiasis.

Characteristics		N ^1^	Good Knowledge ^2^	OR ^3^ (95% CI)	AOR ^4^ (95% CI)
Radio					
	No	523 (94.75)	151 (28.87)	Ref.	Ref.
	Yes	29 (5.25)	12 (41.38)	1.47 (0.67–3.24)	1.09 (0.45–2.63)
Television					
	No	391 (70.83)	109 (27.88)	Ref.	Ref.
	Yes	161 (29.17)	54 (33.54)	1.26 (0.84–1.89)	1.15 (0.71–1.84)
Book/leaflet					
	No	407 (73.73)	112 (27.52)	Ref.	Ref.
	Yes	145 (26.27)	51 (35.17)	1.38 (0.90–2.10)	1.20 (0.75–1.92)
Community leader					
	No	524 (94.93)	154 (29.39)	Ref.	Ref.
	Yes	28 (5.07)	9 (32.14)	0.99 (0.42–2.30)	0.86 (0.34–2.19)
Public health personnel					
	No	185 (33.51)	36 (19.46)	Ref.	Ref.
	Yes	367 (66.49)	127 (34.60)	2.22 (1.43–3.43)	2.18 (1.39–3.43)
Family member					
	No	537 (97.28)	162 (30.17)	Ref.	Ref.
	Yes	15 (2.72)	1 (6.67)	0.18 (0.02–1.43)	0.07 (0.005–1.01)
Neighbor					
	No	546 (98.91)	161 (29.49)	Ref.	Ref.
	Yes	6 (1.09)	2 (33.33)	1.40 (0.23–8.48)	5.24 (0.39–69.95)

^1^ Total sample of 552; ^2^ total with good knowledge, 163; ^3^ odds ratio (OR); ^4^ adjusted odds ratio (AOR).

## Data Availability

The data presented in this study are available upon request from the corresponding author.

## Supplementary Materials

The following supporting information can be downloaded at: https://www.mdpi.com/article/10.3390/tropicalmed7020033/s1. Table S1: Village health volunteers’ reported knowledge of soil-transmitted helminthiasis. Table S2: Village health volunteers’ reported attitudes regarding soil-transmitted helminthiasis. Table S3: Village health volunteers’ reported practices in relation to soil-transmitted helminthiasis.
