# Supplementary material for: Knowledge, Attitudes, and Practices regarding Soil-Transmitted Helminthiasis among Village Health Volunteers in Nakhon Si Thammarat Province, Thailand: A Cross-Sectional Study
Source: Trop Med Infect Dis. 2022 Feb 19;7(2):33. doi: 10.3390/tropicalmed7020033 (PMC8877968; doi:10.3390/tropicalmed7020033)
Supplement: Supplementary file 1 [file tropicalmed-07-00033-s001.zip › tropicalmed-1594550-supplementary.pdf]

Tropical Medicine and Infectious Disease

MDPI

Supplementary Materials

Table S1. Knowledge of soil-transmitted helminthiasis reported by the village health volunteers.

Knowledge items		Total (n = 552)		
		Yes	No	Not sure
**Hookworm**				
1.	The larvae stage of hookworms lives in the soil.	407 (79.7)	39 (7.1)	106 (19.2)
2.	When hookworm larvae enter into the body, they live in the stomach.	397 (71.9)	31 (5.6)	124 (22.5)
3.	Hookworm larvae are abundant in the rainy season.	337 (61.1)	42 (7.6)	173 (31.3)
4.	Hookworm infection can cause anemia.	375 (67.9)	22 (4.0)	155 (28.1)
5.	Hookworm disease is prevented by always defecating in the toilet.	506 (91.7)	13 (2.3)	33 (6.0)
Trichuris trichiura				
6.	Trichuriasis is transmitted by eating food contaminated with the infective stage of eggs.	315 (57.1)	15 (2.7)	222 (40.2)
7.	Symptoms of trichuriasis include throbbing pain, blood in the stool, and colon bulging out of the anus.	229 (41.5)	77 (13.9)	246 (44.6)
8.	The eggs of *Trichuris trichiura* are released from the body through saliva.	124 (22.5)	167 (30.2)	261 (47.3)
9.	Washing fresh vegetables thoroughly before eating can help prevent trichuriasis	398 (72.1)	14 (2.5)	140 (25.4)
10.	Wearing shoes while walking can help prevent trichuriasis.	394 (71.4)	48 (8.7)	110 (19.9)
Ascaris lumbricoides				
11.	Ascariasis is caused by penetration of larvae via the skin.	280 (50.7)	112 (20.3)	160 (29.0)
12.	Symptoms of ascariasis are frequent abdominal pain, bloating, nausea, and vomiting.	407 (73.7)	15 (2.7)	130 (23.6)

	**Knowledge items**	**Total (n = 552)**		
		**Yes**	**No**	**Not sure**
13.	Ascariasis can cause pneumonia and small intestine obstruction.	288 (52.2)	42 (7.6)	222 (40.2)
14.	The eggs of *Ascaris lumbricoides* contaminate vegetables.	296 (53.6)	85 (15.4)	171 (31.0)
15.	Washing fresh vegetables thoroughly before eating can help prevent ascariasis.	390 (70.7)	42 (7.6)	120 (21.7)
	Strongyloides stercoralis			
16.	Larvae of S*trongyloides stercoralis* live in the soil.	339 (61.4)	73 (13.2)	140 (25.4)
17.	Larvae of *Strongyloides stercoralis* can penetrate through the skin.	357 (64.7)	73 (13.2)	122 (22.1)
18.	Symptoms of strongyloidiasis include itching between the fingers.	314 (56.9)	83 (15.0)	155 (28.1)
19.	A blood test can help diagnose strongyloidiasis.	228 (41.3)	86 (15.6)	238 (43.1)
20.	Wearing shoes every time before leaving the house can help prevent strongyloidiasis.	425 (77.0)	43 (7.8)	84 (15.2)

Table S2. Attitudes of soil-transmitted helminthiasis reported by the village health volunteers.

	**Attitudes items**	**Total (n = 552)**		
		**Agree**	**Disagree**	**Not sure**
1.	A person should go for a stool examination for parasites once a year.	543 (98.4)	6 (1.1)	3 (0.5)
2.	If there is a training about soil-transmitted helminthiasis, I will attend the training.	464 (84.1)	9 (1.6)	79 (14.3)
3.	I would like to contribute to prevent soil-transmitted helminthiasis in the community.	481 (87.1)	8 (1.5)	63 (11.4)
4.	Eating unsanitary vegetables can cause a parasitic disease that can be transmitted through soil.	433 (78.5)	41 (7.4)	78 (14.1)
5.	Soil-transmitted helminthiasis can cause anemia.	336 (60.9)	27 (4.9)	189 (34.2)

	**Attitudes items**	**Total (n = 552)**		
		**Agree**	**Disagree**	**Not sure**
6.	Parents play an important role in preventing parasites infection in children.	482 (87.3)	10 (1.8)	60 (10.9)
7.	Some soil-transmitted helminthiasis can be fatal.	307 (55.6)	36 (6.5)	209 (37.9)
8.	Children are more likely to contract helminths that are transmitted through soil than adults.	484 (87.7)	25 (4.5)	43 (7.8)
9.	Soil-transmitted helminthiasis can be prevented.	496 (89.9)	11 (2.0)	45 (8.1)
10.	Adults are less likely to get soil-transmitted helminthiasis than children because they have more immunity to parasites.	284 (51.4)	124 (22.5)	144 (26.1)
11.	Prevention of soil-transmitted helminthiasis is the responsibility of public health officials only.	96 (17.4)	360 (65.2)	96 (17.4)
12.	Walking barefoot on soil lowers the chance of contracting helminths that are transmitted through soil.	106 (19.2)	385 (69.7)	61 (11.1)
13.	Fresh vegetables should not be washed for a long time because it will cause the loss of nutritional value.	101 (18.3)	396 (71.7)	55 (10.0)
14.	Only highly educated people can prevent soil-transmitted helminthiasis.	67 (12.1)	447 (81.0)	38 (6.9)
15.	Defecation in the toilet helps the spread of soil-transmitted helminthiasis.	507 (91.9)	20 (3.6)	25 (4.5)
16.	Soil-transmitted helminthiasis is usually transmitted within families only.	74 (13.4)	371 (67.2)	107 (19.4)
17.	Parasites cannot live on the ground for long periods.	120 (21.7)	167 (30.3)	265 (48.0)
18.	When parasites are transmitted through soil into the body, the symptoms can be clearly seen.	146 (26.5)	142 (25.7)	264 (47.8)
19.	Washing your hands after going to the toilet has no effect on the spread of helminths.	207 (37.5)	250 (45.3)	95 (17.2)
20.	Soil-transmitted helminthiasis can be easily treated with anthelmintic drugs. It is not necessary to prevent infection in any way.	142 (25.7)	281 (50.9)	129 (23.4)
21.	To control soil-transmitted helminthiasis, all members should be given an annual deworming medication.	458 (83.0)	46 (8.3)	48 (8.7)

Table S3. Practices of soil-transmitted helminthiasis reported by the village health volunteers.

	Practices items	Total (n = 552)		
		Usually	Sometime	Never
1.	Wash your hands before eating.	368 (66.7)	184 (33.3)	0 (0)
2.	Wash your hands before cooking.	419 (75.9)	132 (23.9)	1 (0.2)
3.	Wash your hands after defecation.	513 (92.9)	37 (6.7)	2 (0.4)
4.	Wash fresh vegetables thoroughly before eating.	196 (35.5)	345 (62.5)	11 (2.0)
5.	Consume food that has been freshly prepared.	517 (93.7)	35 (6.3)	0 (0)
6.	Keep clothes clean.	481 (87.1)	70 (12.7)	1 (0.2)
7.	Learn about soil-transmitted helminthiasis.	379 (68.7)	169 (30.6)	4 (0.7)
8.	Maintain the cleanliness of the restrooms.	445 (80.6)	99 (17.9)	8 (1.5)
9.	Drink boiled and filtered water.	150 (27.2)	313 (56.7)	89 (16.1)
10.	Keep your nails short.	355 (64.3)	160 (29.0)	37 (6.7)
11.	Biting your nails or sucking your fingers.	532 (96.4)	10 (1.8)	10 (1.8)
12.	Touching pets such as dogs and cats.	29 (5.3)	80 (15.5)	443 (80.2)
13.	Wash your hands after touching pets.	28 (5.1)	248 (44.9)	276 (50.0)
14.	Defecation in a hygienic toilet.	494 (89.5)	55 (10.0)	3 (0.5)
15.	Defecation by digging a hole in the ground.	134 (24.3)	376 (68.1)	42 (7.6)
16.	Defecate on the ground when going out to work in the garden.	430 (77.9)	99 (17.9)	23 (4.2)

	**Practices items**	**Total (n = 552)**		
		**Usually**	**Sometime**	**Never**
17.	Wear shoes when going out.	86 (15.6)	386 (69.9)	80 (14.5)
18.	Wear closed-toed shoes when going out.	36 (6.5)	120 (21.8)	396 (71.7)
19.	Wear boots when leaving the house or going to work in the garden, farm, or field.	14 (2.5)	5 (0.9)	533 (96.6)
20.	Walk barefoot on the ground when going to work in the garden or farm.	42 (7.6)	285 (51.6)	255 (40.8)
